# Tales from the future—nuclear cardio-oncology, from prediction to diagnosis and monitoring

**DOI:** 10.1093/ehjci/jead168

**Published:** 2023-07-19

**Authors:** Nidaa Mikail, Renata Chequer, Alessio Imperiale, Alexander Meisel, Susan Bengs, Angela Portmann, Alessia Gimelli, Ronny R Buechel, Cathérine Gebhard, Alexia Rossi

**Affiliations:** Department of Nuclear Medicine, University Hospital Zurich, Rämistrasse 100, 8091 Zurich, Switzerland; Center for Molecular Cardiology, University of Zurich, Wagistrasse 12, 8952 Schlieren, Switzerland; Department of Nuclear Medicine, Bichat University Hospital, AP-HP, University Diderot, 75018 Paris, France; Nuclear Medicine, Institut de Cancérologie de Strasbourg Europe (ICANS), University Hospitals of Strasbourg, 67093 Strasbourg, France; Molecular Imaging-DRHIM, IPHC, UMR 7178, CNRS/Unistra, 67093 Strasbourg, France; Department of Nuclear Medicine, University Hospital Zurich, Rämistrasse 100, 8091 Zurich, Switzerland; Kantonsspital Glarus, Burgstrasse 99, 8750 Glarus, Switzerland; Department of Nuclear Medicine, University Hospital Zurich, Rämistrasse 100, 8091 Zurich, Switzerland; Center for Molecular Cardiology, University of Zurich, Wagistrasse 12, 8952 Schlieren, Switzerland; Department of Nuclear Medicine, University Hospital Zurich, Rämistrasse 100, 8091 Zurich, Switzerland; Center for Molecular Cardiology, University of Zurich, Wagistrasse 12, 8952 Schlieren, Switzerland; Imaging Department, Fondazione CNR/Regione Toscana Gabriele Monasterio, Via G. Moruzzi 1, 56124 Pisa, Italy; Department of Nuclear Medicine, University Hospital Zurich, Rämistrasse 100, 8091 Zurich, Switzerland; Department of Nuclear Medicine, University Hospital Zurich, Rämistrasse 100, 8091 Zurich, Switzerland; Center for Molecular Cardiology, University of Zurich, Wagistrasse 12, 8952 Schlieren, Switzerland; Department of Cardiology, University Hospital Inselspital Bern, Freiburgstrasse 18, 3010 Bern, Switzerland; Department of Nuclear Medicine, University Hospital Zurich, Rämistrasse 100, 8091 Zurich, Switzerland; Center for Molecular Cardiology, University of Zurich, Wagistrasse 12, 8952 Schlieren, Switzerland

**Keywords:** cardio-oncology, nuclear cardiology, PET, scintigraphy, FDG, myocardial perfusion imaging, CMR, echocardiography, CTRCD

## Abstract

Cancer and cardiovascular diseases (CVD) often share common risk factors, and patients with CVD who develop cancer are at high risk of experiencing major adverse cardiovascular events. Additionally, cancer treatment can induce short- and long-term adverse cardiovascular events. Given the improvement in oncological patients’ prognosis, the burden in this vulnerable population is slowly shifting towards increased cardiovascular mortality. Consequently, the field of cardio-oncology is steadily expanding, prompting the need for new markers to stratify and monitor the cardiovascular risk in oncological patients before, during, and after the completion of treatment. Advanced non-invasive cardiac imaging has raised great interest in the early detection of CVD and cardiotoxicity in oncological patients. Nuclear medicine has long been a pivotal exam to robustly assess and monitor the cardiac function of patients undergoing potentially cardiotoxic chemotherapies. In addition, recent radiotracers have shown great interest in the early detection of cancer-treatment-related cardiotoxicity. In this review, we summarize the current and emerging nuclear cardiology tools that can help identify cardiotoxicity and assess the cardiovascular risk in patients undergoing cancer treatments and discuss the specific role of nuclear cardiology alongside other non-invasive imaging techniques.

## Graphical Abstract

**Graphical Abstract:**
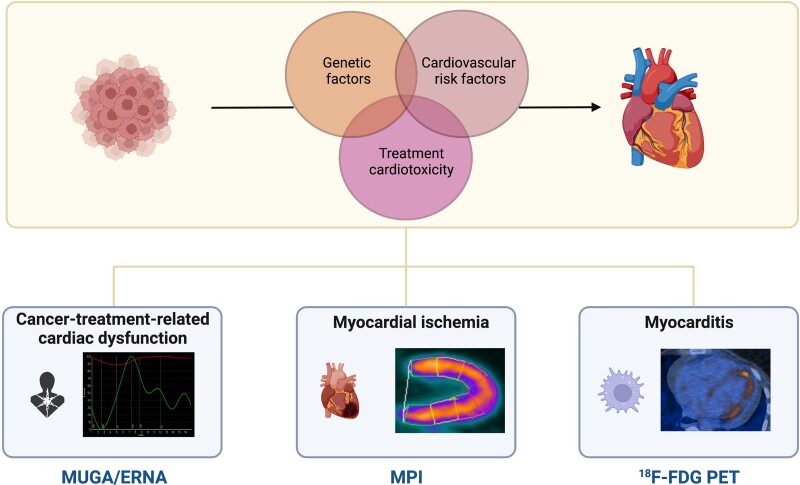
Pathophysiological pathways interconnecting cancers and CVD: genetic predispositions, cardiovascular risk factors, and cancer-treatment-related cardiotoxicity. CVD in cancer patients (and corresponding nuclear cardiology tools) consist mainly of cancer-treatment-related cardiac dysfunction (explored with MUGA/ERNA), myocardial ischaemia (with nuclear MPI), and myocarditis (with ^18^F-FDG PET). Abbreviations: ^18^F-FDG, fluor-18-radiolabelled fluorodeoxyglucose; CVD, cardiovascular diseases; ERNA, equilibrium radionuclide angiography; MPI, myocardial perfusion imaging; MUGA, multigated acquisition; PET, positron emission tomography.

## Introduction

Cancer and cardiovascular diseases (CVD), leading mortality causes in high-income countries,^1^ are interconnected by common pathophysiological mechanisms^2^ and risk factors.^3,4^ Consequently, patients with cancer have an increased risk of CVD and major adverse cardiovascular events (MACE). Vice versa, cardiovascular risk factors (CVRFs) increase cancer risk.^5–7^ Additionally, cancer treatments induce short- and long-term cardiotoxicity.^8,9^ The prognostic improvement of oncological patients is slowly shifting their burden from cancer to cardiovascular mortality.^10^ Hence, cardio-oncology is a steadily expanding field, as evidenced by the recent publication of the first European Society of Cardiology (ESC) cardio-oncology guidelines,^11^ prompting the need for cardiovascular risk stratification markers in oncological patients.^12,13^ Despite being challenged by echocardiography and cardiac magnetic resonance (CMR),^14^ nuclear imaging remains a contemporary modality in patients receiving cardiotoxic therapies.

In this article, we briefly summarize the central mechanisms responsible for cancer-treatment-induced cardiotoxicity, review the main established and emergent nuclear cardiology tools useful in cancer settings, and discuss the role of nuclear medicine alongside echocardiography and CMR. Although also beneficial for managing cardiac tumours,^15,16^ this review will not cover this topic.

## Mechanisms of interaction between cancer and CVD

**Figure 1:**
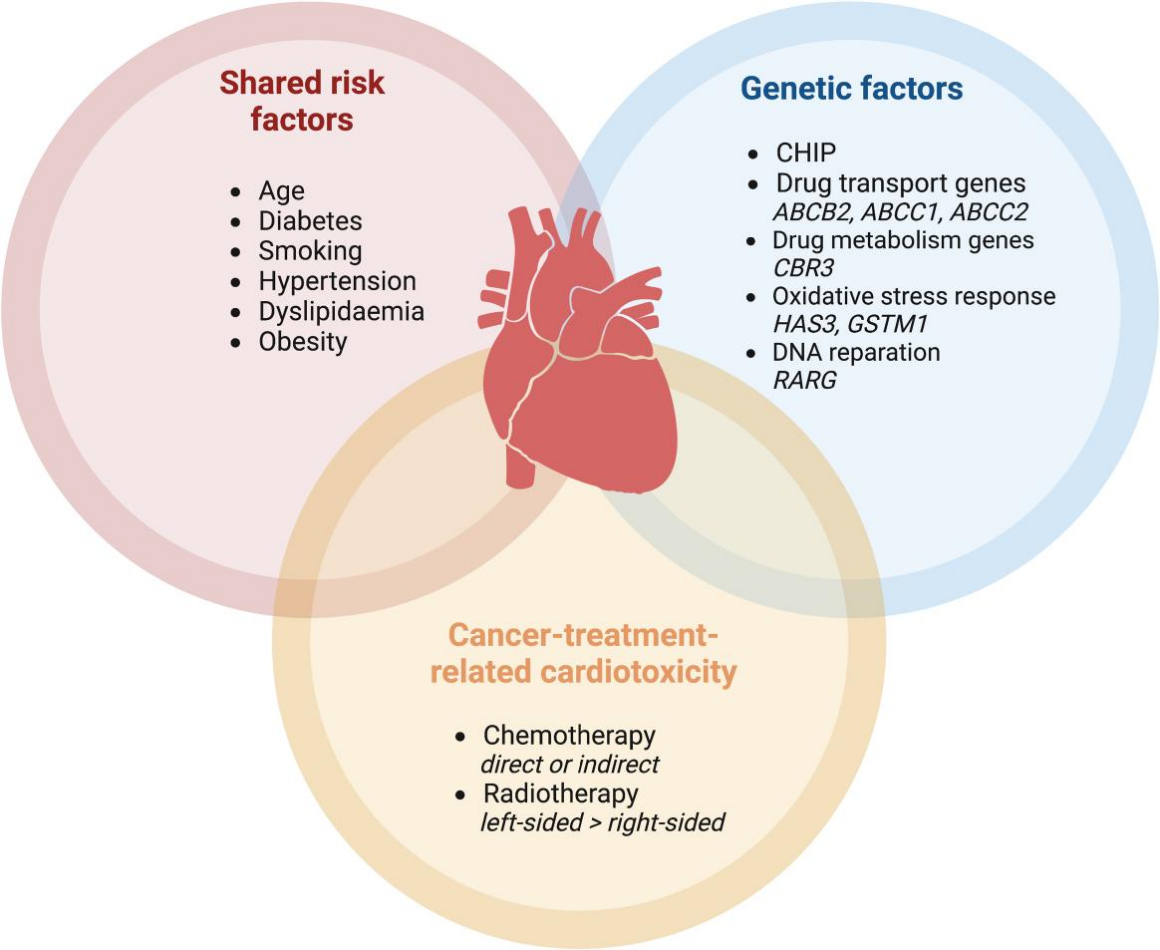
Mechanisms of CVD in cancer patients. Abbreviations: ABCB2, ABC transporter B family member 2 gene; ABCC1, ATP-binding cassette subfamily C member 1 gene; ABCC2, ATP-binding cassette subfamily C member 2 gene; CBR3, carbonyl reductase 3 gene; CHIP, clonal haematopoiesis of indeterminate potential; GSTM1, glutathione S-transferase mu 1 gene; HAS3, hyaluronan synthase 3 gene; RARG, retinoic acid receptor gene.

CVD and cancer are two sides of the same coin,^17^ sharing identical pathophysiological pathways^18^ (*Figure 1*).

### Risk factors

Typical CVRFs include age, diabetes, hypertension, smoking, dyslipidaemia, and overweight,^19^ all of which concomitantly increase cancer risk.^20^ By promoting inflammation and oxidative stress, diabetes favours a pro-oncogenic environment.^21^ Similarly, epidemiological data suggest a correlation between hypertension and dyslipidaemia on the one hand and cancer genesis on the other.^22^ Smoking promotes atherosclerosis and cancer,^23,24^ and a plethoric adipose tissue triggers oncogenic inflammatory molecules.^25^

### Genetic factors

Intrinsic factors also predispose to CVD and cancer.^26^ For instance, specific age-related somatic mutations, labelled clonal haematopoiesis of indeterminate potential (CHIP), increase the risk of haematological malignancy^27^ and CVD.^28^ Other genes involved in drug delivery and metabolism modulate the risk of cancer-therapy-induced cardiotoxicity,^26^ either by increasing it, such as ATP-binding cassette transporters *ABCB4* and *ABCC*, or by decreasing it, for example, ATP-binding transporters (*ABCB1*) and solute carriers (*SLC28A3*).^26^

### Cancer-treatment-related cardiotoxicity

Cancer-treatment-induced cardiotoxicity is a critical contributor to CVD^18^ (*Table 1*). Cancer-treatment-related cardiac dysfunction (CTRCD), i.e. left ventricular (LV) dysfunction induced by cancer treatment, is the most common cardiotoxicity type.^8^ Two types of CTRCD are distinguished.^29,30^ Type I CTRCD, classically caused by anthracyclines, induces direct cumulative, dose-related, and usually irreversible cardiomyocyte damage. Type II CTRCD, traditionally induced by trastuzumab,^29^ is a reversible and dose-independent myocardial dysfunction without structural alterations. Cancer treatment can also induce coronary artery disease (CAD), notably vasospasm and arterial thrombosis.^31,32^ Likewise, chest radiotherapy favours atherosclerosis and fibrosis via inflammatory cascades in the coronary vessels.^6,33^

Lately, the introduction of immune checkpoint inhibitors (ICI) to the cancer armamentarium was accompanied by increasing reports of immune-related adverse events (IRAEs),^34,35^ including myocarditis.^36^

**Table 1:** Main types of cancer treatments and related toxic effects

Therapeutic class	Main treatment-induced toxicity mechanisms
Anthracyclines	Induction of oxidative stress, impaired autophagy, type II topoisomerase poisoning
Trastuzumab	Inhibition of epidermal growth factor receptor 2
Fluoropyrimidines	Induction of oxidative stress in cardiomyocytes, vasospasm by favouring endothelial and smooth cell dysfunction, coronary artery thrombosis
Platinum drugs	Induction of oxidative stress and of direct damage to cardiomyocytes and mitochondria, platelet aggregation
Taxanes	Direct cardiomyocyte and mitochondrial damage, alteration of cell division and microtubule dysfunction, oxidative stress, platelet aggregation, endothelial injury, haemorrhagic myopericarditis
Vascular endothelial growth factor (VEGF) inhibitors (tyrosine kinase inhibitors, monoclonal antibodies)	Arterial and venous thrombosis
Immune checkpoint inhibitors	Increased CD4 and CD8 lymphocyte infiltration inducing myopericarditis and arrhythmia
Radiotherapy	Coronary atherosclerosis and fibrosis by triggering acute and long-term coronary inflammation

## Role of imaging for the early detection of CVD in cancer patients

The European Society for Medical Oncology guidelines highlight the need for an early screening of CVRFs and close cardiovascular monitoring of cancer patients.^13^ This assessment includes a baseline evaluation of LV ejection fraction (LVEF) to guide the cancer treatment choice and the need for cardioprotective therapies.^13^ However, LVEF alone can prove insufficient, since an LVEF drop is often a late-stage manifestation of cardiac damage.^37,38^ Global longitudinal strain (GLS) assessment using echocardiography or CMR is a more sensitive marker of cardiac dysfunction and is, therefore, recommended.^14^ Nonetheless, GLS is limited by scarce reproducibility,^39^ prompting the need for alternative tools.

Nuclear medicine imaging and particularly multigated acquisition (MUGA) scintigraphy have historically been at the frontline of LV monitoring in oncological patients.^40,41^ Although challenged by CMR,^42^ nuclear cardiology provides critical information for diagnosing, monitoring, and risk-stratifying cancer patients^15,16,43–45^ (*Table 2*). In the following part, we will review how nuclear cardiology can detect cardiac complications in oncological patients and discuss its role alongside echocardiography and CMR.

**Table 2:** Main types of cancer-treatment-related cardiotoxicities and main cardiac cancers with the corresponding nuclear imaging diagnostic tools

	Type of toxicity/disease	Most common toxic agents	Imaging tools	Comments
Cardiotoxicity	CTRCD	Anthracyclines, alkylating agents, TKI, proteasome inhibitors	MUGA (ERNA SPECT for RV function) ±First-pass ^18^F-FDG PET	Diagnosis and monitoring of LV dysfunction
	Coronary artery disease	Alkylating-like agents, fluoropyrimidine (vasospasm) taxanes, radiotherapy, hormonotherapy (Arimidex, Aromasin, Femara)	SPECT MPI PET MPI	CACS derivable from hybrid CT imaging CMVD with PET MPI LVEF from SPECT and PET MPI
	Myocarditis	Alkylating agents, immune checkpoint inhibitors	^18^F-FDG PET ±^68^Ga-SSTR PET ±^68^Ga-FAPI PET ±^89^Zr-DFO-CD4 and ^89^Zr-DFO-CD8a PET	Potential role for hybrid PET/CMR
Specific disease	Cardiac tumours	NA	^18^F-FDG for aggressive primary tumours and NECs ^68^Ga-SSTR for low-grade NETs	Diagnosis and staging

Abbreviations: ±, optional or used in research studies; ^18^F-FDG, fluor-18-radiolabelled fluorodeoxyglucose; ^68^Ga-FAPI, gallium-68-radiolabelled fibroblast activation protein inhibitors; ^68^Ga-SSTR, gallium-68-radiolabelled somatostatin receptor; ^89^Zr-DFO-CD4, zirconium-89-radiolabelled desferrioxamine-CD4; ^89^Zr-DFO-CD8a, zirconium-89-radiolabelled desferrioxamine-CD8a; ^99m^Tc, technetium-99m; ^123^I-MIBG, iodine-123 metaiodobenzylguanidine; ATTR, transthyretin amyloidosis; CA, cardiac amyloidosis; CACS, coronary artery calcium score; CMVD, coronary microvascular dysfunction; CMR, cardiac magnetic resonance; CT, computed tomography; CTRCD, cancer-treatment-related cardiac dysfunction; ERNA, equilibrium radionuclide angiography; LVEF, left ventricular ejection fraction; MPI, myocardial perfusion imaging; MUGA, multigated acquisition; NA, not applicable; NEC, neuroendocrine carcinoma; NET, neuroendocrine tumour; PET, positron emission tomography; SPECT, single-photon emission computed tomography; TKI, tyrosine kinase inhibitors.

## Diagnosis of cancer-treatment-related toxicity

### CTRCD and LV systolic dysfunction

**Figure 2:**
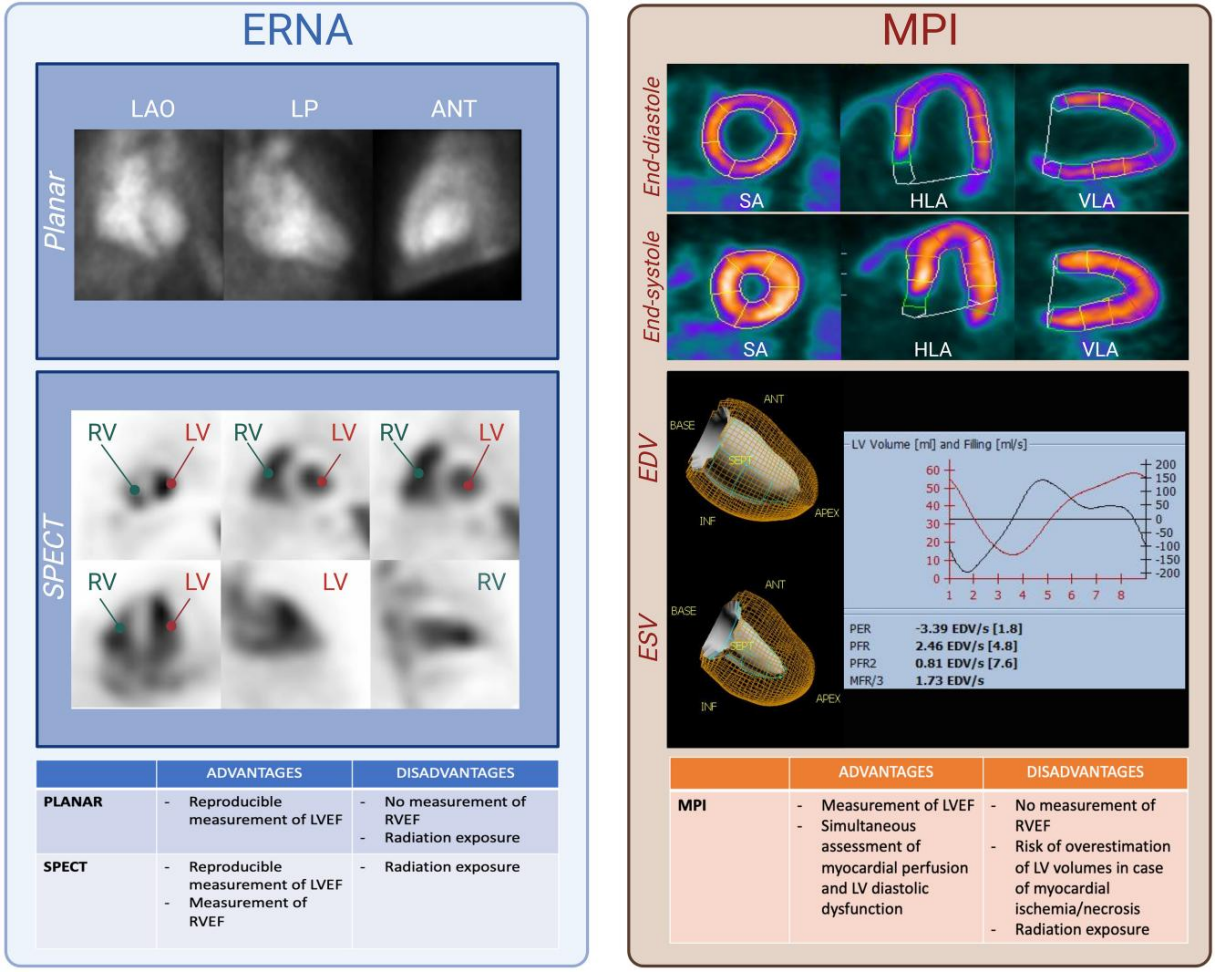
LV function assessment with nuclear cardiology. *Left panel*: ERNA techniques for LVEF assessment based on radiolabelled erythrocytes’ activity. Planar ERNA: end-diastolic and end-systolic LV volumes derived from LAO projections. Additional incidences include LP and anterior projections. SPECT ERNA: 3D reconstructions allowing LVEF/RVEF measurement. *Right panel*: NH_3_ PET MPI during end-diastole and end-systole enabling EDV/ESV estimation. Accurate volume measurement with MPI necessitates preserved myocardial perfusion. Diastolic (D) function can also be studied. Abbreviations: ANT, anterior; CHIP, clonal haematopoiesis of indeterminate potential; EDV, end-diastolic volume; ERNA, equilibrium radionuclide angiography; ESV, end-systolic volume; HLA, horizontal long axis; LAO, left anterior oblique; LP, left profile; LV, left ventricle; LVEF, left ventricular ejection fraction; MFR, mean filling rate during the first third of diastole; mL, millilitres; mL/s, millilitres per second; MPI, myocardial perfusion imaging; PER, peak ejection rate; NH_3_, ammonium; PET, positron emission tomography; PFR, peak filling rate; PFR/2, peak filling rate during the first half of diastole; RV, right ventricle; RVEF, right ventricular ejection fraction; SA, short axis; SPECT, single-photon emission computed tomography; VLA, vertical long axis.

**Figure 3:**
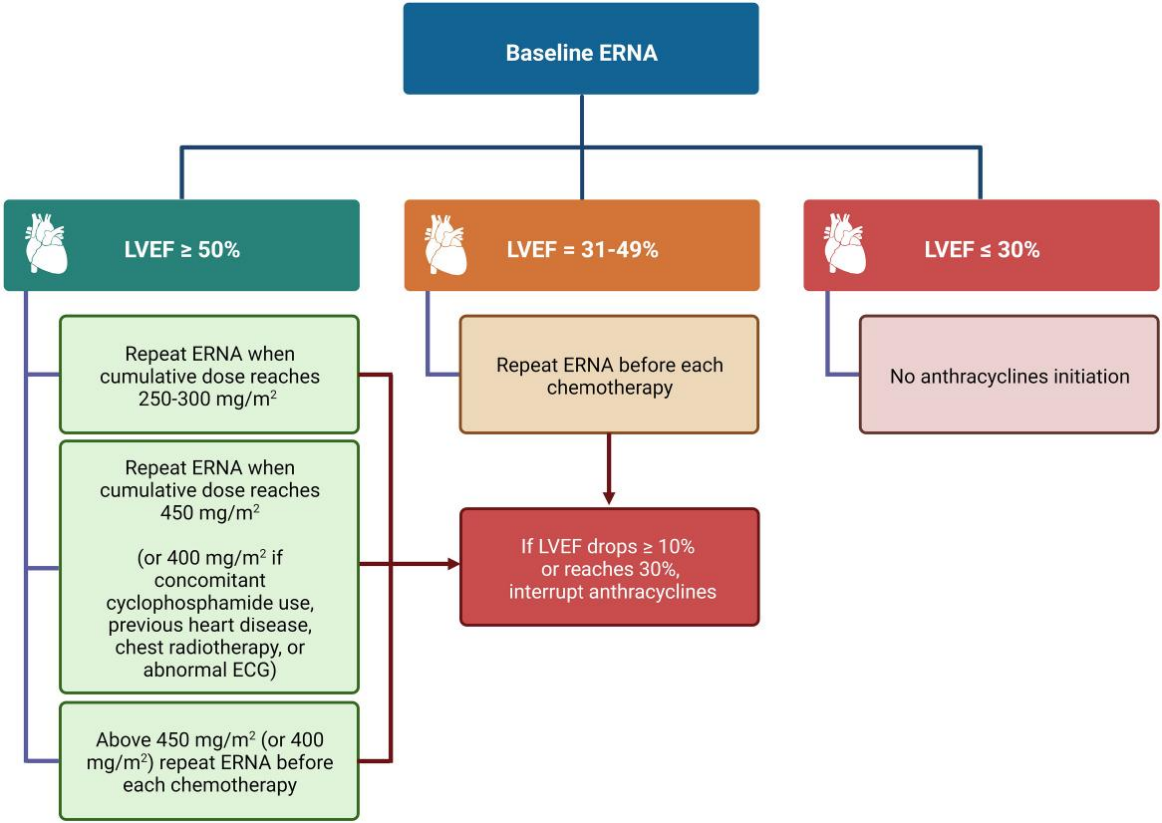
SNMMI/EANM Guidelines for ERNA-based LVEF monitoring in anthracycline-treated patients. Abbreviations: EANM, European Association of Nuclear Medicine; ECG, electrocardiogram; ERNA, equilibrium radionuclide angiography; LVEF, left ventricular ejection fraction; SNMMI, Society of Nuclear Medicine and Molecular Imaging.

The ESC defines CTRCD as (i) a ≥10% LVEF decrease from baseline to below 50%, (ii) with a GLS drop of ≥15% from baseline, confirmed by a 2–3-week repeat study, in the context of cancer treatment.^14^ While only echocardiography and CMR can estimate GLS,^46^ MUGA robustly determines LVEF.^47,48^ In MUGA, cardiac volumes are derived from heart-centred images of the patient’s own radiolabelled erythrocytes^49^ and are therefore not influenced by geometric assumptions about the myocardial wall.^50^ Three types of MUGA are distinguished: (i) first-pass MUGA, (ii) planar equilibrium radionuclide angiography (ERNA), and (iii) single-photon emission computed tomography (SPECT) ERNA. In practice, first-pass MUGA is limited to specific indications [right ventricular ejection fraction (RVEF) and shunt assessment^49,51^], and only ERNA is used to assess CTRCD. Planar ERNA is acquired when the radiotracer has reached equilibrium and allows measuring LVEF^52^ (*Figure 2*), not RVEF, because of the superposition of heart structures. However, ERNA can also be performed with three-dimensional (3D) gated SPECT, which enables the delineation of both LVEF and RVEF.^49,53–56^ Overall, MUGA displays a high inter- and intra-observer reproducibility for LVEF measurement,^57^ which is crucial for serial follow-up during anticancer treatment.^14,58^ MUGA also helps select patients who can safely tolerate higher cumulative anthracycline doses, i.e. asymptomatic patients with LVEF > 40% and a drop in LVEF < 10%,^13,41^ significantly reducing heart failure occurrence.^59^ Although in good agreement,^60^ LVEF tends to be higher with SPECT than with planar ERNA,^61^ which needs to be taken into consideration for monitoring.^14^ Similarly, in breast cancer patients, MUGA gives slightly lower LVEF values than CMR.^62^ As such, when using MUGA, for an LVEF threshold of 50%, this difference could result in 35% more patients being diagnosed with CTRCD than with CMR.^62^ Hence, given that LV volumes tend to shrink and LVEF to increase after menopause,^63^ CTRCD thresholds might need to be adapted in women.^64^ Regarding surveillance, the European and American nuclear medicine societies recently issued an expert consensus for monitoring LVEF by ERNA for patients receiving anthracyclines,^65^ which has been summarized in *Figure 3*.

A major drawback of MUGA is radiation exposure. Indeed, MUGA requires the injection of 555–1110 MBq (7–15 MBq/kg in children) of radiotracers,^65^ which in case of serial follow-up increases theoretically (albeit minimally) cancer risk.^66–68^ Cadmium-zinc-telluride (CZT)-based cameras, which detectors are more sensitive than conventional sodium iodide (NaI) ones, enable a two- to three-fold reduction in injected activity without altering image quality,^60,69,70^ hence decreasing the radiation burden. CZT-derived LVEF highly correlates with the one obtained from planar NaI detectors.^71^ CZT-based SPECT ERNA is also in high agreement with CMR for RVEF.^72^ Interestingly, LVEF can be obtained from fluor-18-radiolabelled fluorodeoxyglucose positron emission tomography (^18^F-FDG PET) gated first-pass acquisitions, showing excellent concordance with planar ERNA.^73^ Given that ^18^F-FDG PET is the mainstay for cancer staging, this elegant approach allows simultaneously measuring LVEF with no additional radiation exposure. However, first-pass cardiac ^18^F-FDG acquisitions result in a prolonged acquisition time (5 min), reducing the available scanning time for other patients.

**Figure 4:**
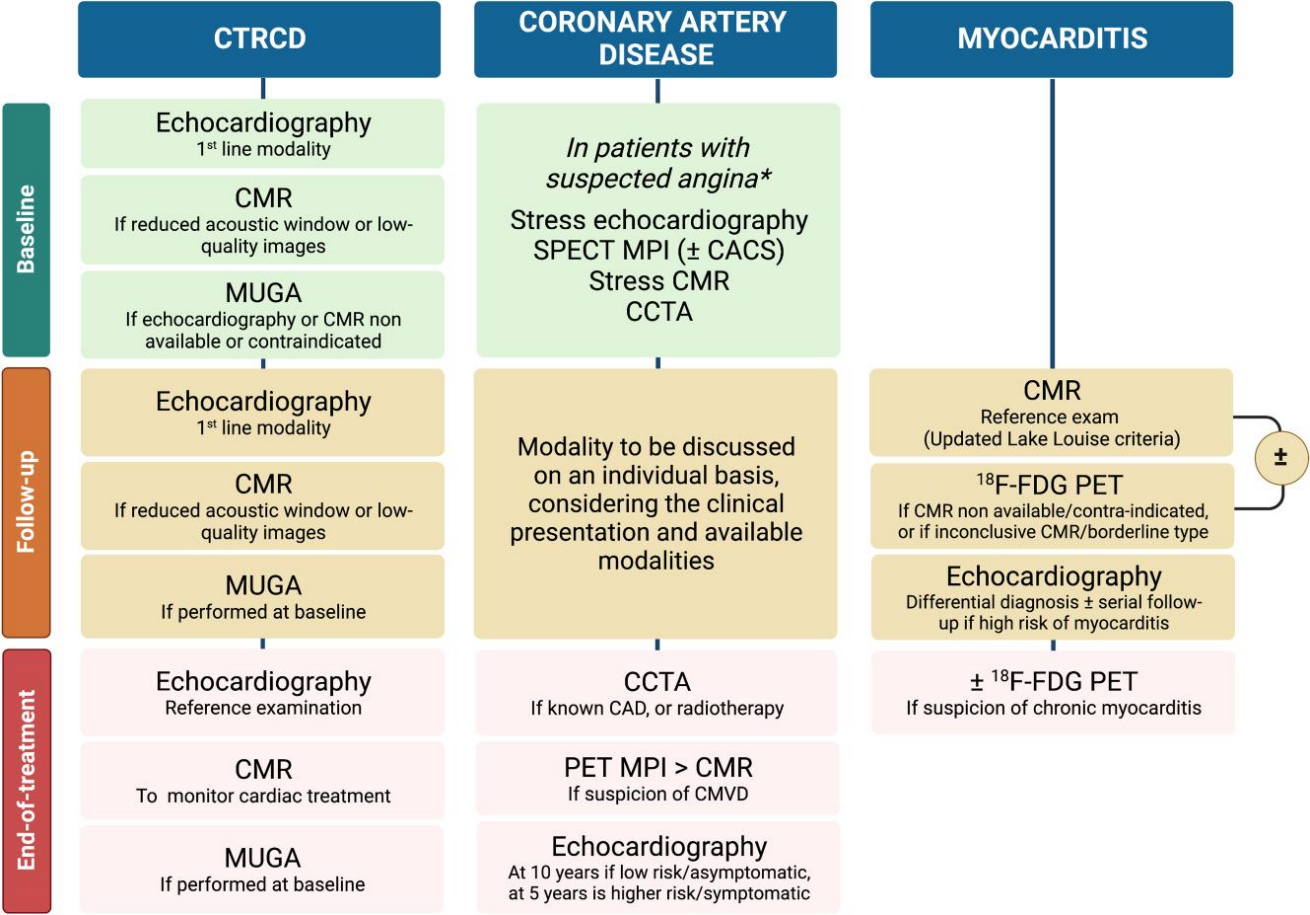
Algorithm proposal for non-invasive imaging in patients undergoing anticancer treatment. Abbreviations: ^18^F-FDG, fluor-18-radiolabelled fluorodeoxyglucose; CACS, coronary artery calcium score; CAD, coronary artery disease; CCTA, coronary computed tomography angiography; CMR, cardiac magnetic resonance; CTRCD, cancer-treatment-related cardiac dysfunction; LVEF, left ventricular ejection fraction; SNMMI, Society of Nuclear Medicine and Molecular Imaging; MPI, myocardial perfusion imaging; MUGA, multigated acquisition; PET, positron emission tomography; SPECT, single-photon emission computed tomography. *The choice of imaging modality should be based on symptoms, known CAD, pretest probability, local availability and expertise, and patient characteristics.

In practice, MUGA has long been supplanted by the more readily available and non-irradiating echocardiography and CMR (*Figure 4*). Transthoracic echocardiography (TTE) is the frontline risk stratification exam, owing to its wide availability, harmfulness, ability to assess morphology (including valves), function, and GLS. Whenever available, 3D echocardiography is preferred over 2D, given its higher reproducibility for LVEF and GLS assessment.^74–76^ GLS detects early signs of systolic dysfunction before any LVEF drop, with a change in GLS ≥ 15% predicting the risk of CTRCD.^46^ Importantly, a GLS-based cardioprotective strategy reduces the rate of CTRCD in patients undergoing anthracycline.^77^ Nonetheless, echography strain measurements lack inter-device standardization, which limits their routine use.^78^ In case of reduced acoustic window or low image quality, CMR is recommended as a second-line technique.^11,75^ CMR is considered the reference exam to calculate cardiac volumes and function and can detect even minor LVEF impairments and volume changes.^75^ The latter is particularly important in patients undergoing anticancer treatments, in whom CTRCD can manifest as an isolated LV end-diastolic volume reduction.^79^ Moreover, CMR accurately determines RVEF, which can be asymptomatically reduced in cancer survivors.^80^ Besides volumes and strain assessment, CMR is a promising tool for the early detection of cancer-treatment-related myocardial oedema and fibrosis via T1/T2 mapping and extracellular volume (ECV) measurement.^81^ Increased T1/T2 relaxation times hold promise to predict subsequent CTRCD,^81^ although there is a significant overlap between mapping parameters of patients who develop CTRCD and those who do not.^82^

During cancer treatment, echocardiography is the preferred modality for monitoring cardiac function.^14^ Surveillance frequency depends on a cardiotoxicity risk profile based on patient- and treatment-related factors.^14^ Importantly, given the inter-imaging variability, it is crucial to perform follow-up using the same modality.^83^ Indeed, minor LVEF variations are essential to detect, as they could be an early sign of cardiac toxicity. Compared with CMR, 2D and 3D TTE tend to underestimate LV volumes.^84^ Similarly, limits of agreement between MUGA and CMR often exceed ±10%,^48^ which could lead to incorrectly classifying patients with CTRCD. In this regard, MUGA’s radiation exposure argues against its systematic use for the follow-up of patients undergoing anticancer treatment.

After the end of treatment, patients who developed CTRCD should be monitored using echocardiography. In patients in whom a cardiac medication was introduced to mitigate treatment side effects, CMR is an option to assess treatment response.^14^

In summary, the 2022 ESC Guidelines on cardio-oncology only recommend MUGA as a third-line technique to assess LVEF, i.e. if TTE and CMR are unavailable or in case of CMR contraindication.^11,14^ Of note, the guidelines mention the potential interest of assessing the myocardial ^18^F-FDG uptake during intercourse PET/computed tomography (CT), as its increase could indicate an LVEF decline^85^ and, therefore, trigger LVEF assessment.^14^

### Coronary artery disease

Cancer is a prothrombotic condition associated with enhanced platelet reactivity and circulating procoagulant products, which increase the atherosclerotic burden.^86^ Additionally, cancer treatments themselves (particularly chest radiotherapy) induce endothelial injuries, favouring vasospasm and thrombosis.^87–89^ Hence, screening for ischaemic heart diseases (IHD) is recommended in patients with intermediate-to-high pre-test likelihood^46^ undergoing heart-damaging cancer therapy,^8^ especially anthracyclines and chest radiotherapy.^90,91^ Such screening can be done with SPECT myocardial perfusion imaging (MPI), a mainstay in this indication.^92^

SPECT myocardial perfusion abnormalities can appear either during radiotherapy^93^ or later, up to 20 years after treatment completion.^94^ Most perfusion abnormalities develop in the apical territory,^89,95^ indicating left anterior descending artery damage.^96,97^ Accordingly, myocardial perfusion impairment is more prevalent in left-sided than right-sided chest cancer,^89,98^ a risk that linearly correlates with cardiac exposure volume.^94,99^ In patients with left-sided breast cancer, an irradiated cardiac volume of >5% is associated with significantly higher rates of perfusion abnormalities than with lower volumes.^100^ Interestingly, in cancer patients, SPECT-detected myocardial ischaemia does not correlate well with underlying obstructive CAD,^97^ highlighting the importance of coronary microvascular dysfunction (CMVD) and coronary spasm in this population.^101–103^

PET MPI is the reference non-invasive modality to diagnose CMVD, using ^13^N-ammonia (^13^N-NH_3_), ^82^Rubidium (^82^Rb), and ^15^O-water (^15^O-H_2_O) radiotracers.^104^ PET MPI allows for measuring myocardial blood flow (MBF) and coronary flow reserve (CFR), which are central to CMVD diagnosis.^105^ In patients undergoing chest radiotherapy, PET MPI shows an inverse correlation between the mean radiation dose to the heart and CMVD.^106,107^ Moreover, MBF could have prognostic value, with low CFR values being associated with an increased cumulative incidence of MACE in breast cancer patients.^108^

Another prognostic parameter is the coronary artery calcium score (CACS). CACS is obtained from a non-enhanced CT and quantifies the degree of coronary artery calcification, expressed with the Agatston score.^109^ CACS = 0 in asymptomatic patients is associated with a very low prevalence of severe coronary stenosis and high-risk plaque features.^110^ Conversely, an Agatston score of >400 is predictive of MACE, even for normal MPI.^111^ CACS can easily be yielded from the low-dose CT of PET/CT and SPECT/CT cameras, showing high agreement with the one obtained from standard non-enhanced scans.^112–114^ Since ^18^F-FDG PET/CT is part of routine oncological work-up, cardiovascular risk stratification with CACS could simultaneously be performed without additional radiation or cost.^115^

Beyond myocardial perfusion, nuclear MPI can also estimate LVEF^116,117^ (*Figure 2*). However, contrary to MUGA, MPI indirectly estimates cardiac volumes based on myocardial wall motion. In case of infarction, the necrotic segment is devoid of signal, leading to overestimating LV volumes.^118^ Another limitation of SPECT MPI is its inability to assess RVEF. Although more accurate,^119–121^ CZT cameras give lower values than conventional SPECT cameras,^122^ stressing the importance of performing serial follow-up using the same modality.

Nuclear MPI is only one of the tools available for myocardial ischaemia screening alongside stress echocardiography, and CMR. Additionally, contrast-enhanced coronary computed tomography angiography (CCTA) is an alternate tool which provides information on coronary plaque burden and coronary stenosis assessment.^11,14^ Although the recent European guidelines on cardio-oncology do not give strict recommendations on which modality to prefer in which setting,^11^ echocardiography and CMR remain the frontline techniques in this setting.^14^ Overall, three scenarios can be distinguished: baseline screening, follow-up during treatment, and end-of-treatment surveillance^14^ (*Figure 4*).

Baseline screening should always be considered in the oncological population, given their increased CAD risk.^14^ CACS assessment is an easy and minimally invasive way of characterizing the baseline CAD risk. If CACS = 0, the risk of dying from CAD within 5 years of cancer diagnosis remains below the mortality risk from cancer itself; conversely, if CACS > 300, the 5-year CAD mortality risk exceeds the cancer mortality risk,^123^ prompting more aggressive management.^14^ As abovementioned, CACS can be extracted from ^18^F-FDG PET’s low-dose CT without additional scanning time, cost, or radiation.^115^ As the mainstay baseline staging exam of most cancer types, ^18^F-FDG-PET-based CACS appears as a reasonable option for baseline CAD risk assessment. Advanced explorations should be preferred in patients with a higher baseline CAD risk. In nononcological settings, non-enhanced CT and CCTA are the first-line exam for detecting coronary calcifications and coronary stenosis in patients with low-to-intermediate CAD risk.^92^ Given the increased CAD risk in oncological patients, detection of coronary stenosis using CCTA can be discussed in symptomatic patients with no CAD history.^14^ However, this comes at the expense of increased radiation exposure.^124^ Stress echocardiography is indicated in patients with intermediate-to-high CAD probability undergoing ischaemia-inducing chemotherapies, such as fluorouracil, bevacizumab, sorafenib, and sunitinib.^125^ In addition to ischaemia, stress echocardiography could unveil patients at risk of developing CTRCD,^126,127^ a 5-unit fall in LV contractile reserve during dobutamine echocardiography predicting the subsequent LVEF drop.^128^ Myocardial perfusion CMR imaging using pharmacological stress is also an option, but its use for systematic screening is conflicted by its relatively low availability.^14^ SPECT MPI is a well-validated and widely accessible modality that additionally provides CACS in case of hybrid SPECT/CT.^113^

During treatment, there is no clear recommendation as to which modality to prioritize and the exploration frequency, which will depend on the clinical presentation and the available modalities.

After treatment completion, CCTA is an option for CAD identification,^14^ particularly in patients with known CAD, whose plaque progression can be accelerated by anticancer treatment, and in young patients treated with chest radiotherapy, i.e. at risk of perivascular fibrosis.^14,90^ Radiotherapy can also induce valve leaflet calcification, which can be assessed by CT.^90^ A limitation of CCTA is for the routine detection of microvascular dysfunction, although dynamic CT MPI is promising in this regard.^129–132^ Conversely, CMR detects both segmental ischaemia and CMVD,^133^ with the advantage over nuclear MPI of being devoid of radiation exposure. Still, CMR assessment of MBF remains in the research realm,^134^ and PET MPI is the reference exam for CMVD,^104^ displaying higher accuracy, reproducibility, and prognostic value than CMR.^135,136^ This favours PET in patients at risk of CMVD, particularly women with breast cancer^108,137^ and patients who underwent chest radiotherapy.^106,138^

### Myocarditis

The last years have witnessed the development of immunotherapy, a new class of anticancer treatment that leverages the immune system to harness cancer progression. The primarily used class of immunotherapy is ICI. Immune checkpoints are T-lymphocyte-expressed receptors that recognize ligands at the surface of normal cells. The receptor–ligand binding inhibits the T-cell, preventing it from targeting normal cells.^139^ Some cancer cells express immune-checkpoint-binding ligands and can thus trick and inhibit T-lymphocytes. ICI block the receptor–ligand bond, allowing T-cells to recognize and attack cancer cells.^139^ The downfall of lifting T-cell inhibition is that this may unleash IRAEs.^140^ Cardiovascular IRAEs occur with an incidence ranging from 1.14 to 5%^140^ and include notably myocarditis, pericarditis, vasculitis, and Takotsubo cardiomyopathy.^141,142^

**Figure 5:**
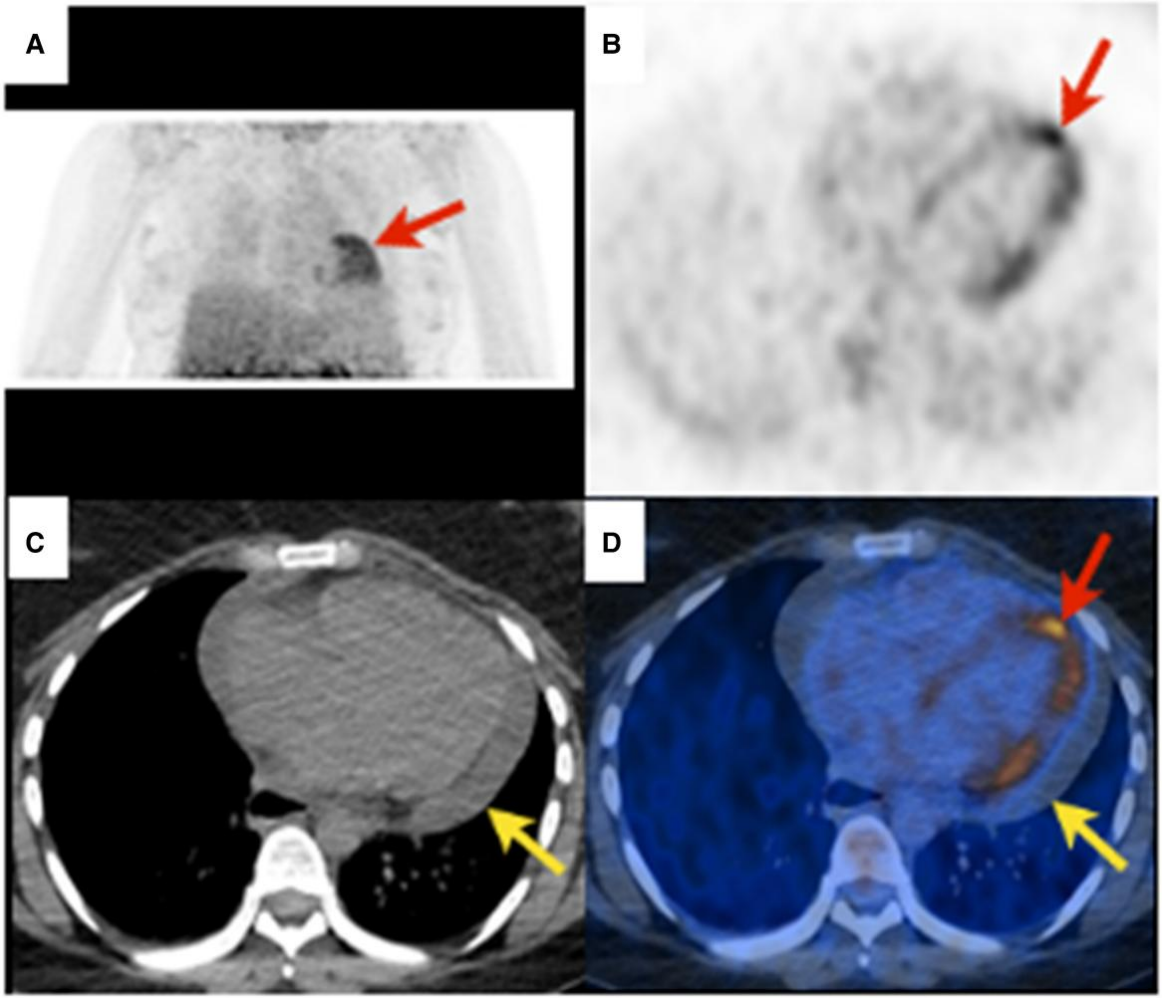
Myocarditis. Twenty-seven-year-old dyspnoeic woman with widespread concave ST elevation on ECG and increased C-reactive protein (131 mg/L, *N* < 4), suggestive of myocarditis. ^18^F-FDG PET revealing diffuse heterogeneous (‘patchy’) myocardial ^18^F-FDG uptake [red arrows, (*A*) maximal intensity projection, (*B*), and (*D*) axial slices]. Non-enhanced CT showing pericardial effusion [(*C*), yellow arrow] without ^18^F-FDG uptake [(*D*), yellow arrow] related to pericarditis. According to the Bonaca *et al.*^147^ criteria, *possible myocarditis* was retained.

Diagnosing ICI-related myocarditis is challenging because of the various presentations^143^ and the prolonged interval between drug administration and symptom onset.^140^ While CMR is the reference exam,^144^ PET can also orient the diagnosis. Due to its availability and high uptake in inflammatory cells, ^18^F-FDG is a natural candidate in this indication,^145^ classically displaying focal or diffuse patchy myocardial ^18^F-FDG uptake with no vascular systematization^146^ (*Figure 5*). Despite a good spatial agreement between ^18^F-FDG uptake and T2 hyperintensity/late gadolinium enhancement (LGE), the diagnostic accuracy of ^18^F-FDG PET/CT in myocarditis is low.^148^ Several factors might explain this, such as an inadequate high-fat/low-carbohydrate diet, the initiation of immunosuppressive treatment, and the delay between myocarditis onset and image acquisition. Acquisition timing is indeed critical, with a small series showing a 100% sensitivity when ^18^F-FDG PET was performed within 14 days of disease onset vs. 20% when performed later.^146^ In 2019, Bonaca *et al.*^147^ proposed a definition of ICI-related myocarditis that includes ^18^F-FDG PET, with myocarditis deemed as *possible* in any ‘scenario meeting criteria for possible myocarditis (i.e. not explained by any other diagnosis such as acute coronary syndrome, trauma or Takotsubo cardiomyopathy on CMR, ultrasound, and cardiac biomarkers) with ^18^F-FDG PET showing patchy cardiac ^18^F-FDG uptake without another explanation’.

Other promising radiotracers target somatostatin receptors (SSTRs) overexpressed at the surface of vascular macrophages.^149^ The lower albeit variable^150^ physiologic myocardial uptake of SSTR radiotracers reduces the risk of false positives. In a small population of nine patients, gallium-68-radiolabelled DOTA0-D-Phe1-Tyr3-octreotide (^68^Ga-DOTATOC) PET/CT had a 100% sensitivity to diagnose ICI-related myocarditis, despite the initiation of steroid and immunosuppressive therapy.^151^ Recently, a ^68^Ga-radiolabelled tracer targeting fibroblast activation protein inhibitors (^68^Ga-FAPI) was introduced in oncological diseases.^152,153^ In three patients fulfilling the Bonaca *et al.*^147^ criteria for definite ICI-related myocarditis, focal myocardial uptake of ^68^Ga-FAPI identified cardiac remodelling territories.^154^ Similarly, the upregulation of translocator protein-18 kDa (TSPO) and chemokine receptors types 4 and 12 in inflammatory cells suggests a role for radiolabelled TSPO and ^68^Ga-pentixafor in myocarditis,^155,156^ although dedicated studies still need to be performed. Along the same line, radiotracers targeting the C-X-C motif chemokine receptor 4 overexpressed by leucocytes represent an exciting approach to diagnosing myocardial inflammation.^157^ Finally, novel inflammation radiotracers targeting CD4 and CD8 cells, zirconium-89-radiolabelled desferrioxamine-CD4 (^89^Zr-DFO-CD4) and ^89^Zr-DFO-CD8a, are under investigation and hold the potential to image myocarditis.^158^ Their high specificity could prove particularly useful in ICI-related myocarditis. Indeed, the reference treatment for myocarditis is steroids, which alleviate the antitumour effect of ICI.^159^ Therefore, establishing the diagnosis with certainty might reduce unnecessary immunosuppressive therapies or withholding ICI.

In practice, however, the guidelines recommend echocardiography and CMR as first-line examinations in suspected ICI-associated myocarditis and recommend cardiac PET only if CMR is non-available or contraindicated^11^ (*Figure 4*). Echocardiography’s primary role is to rule out non-inflammatory cardiac diseases and serve as a reference exam for LVEF monitoring.^160^ Serial echocardiography could also be discussed in patients at high risk of myocarditis, i.e. patients undergoing a combination of ICI, ICI with another cardiotoxic regimen, or in case of pre-existing CVD.^14^ The mainstay examination for diagnosing myocarditis remains CMR, using the Lake Louise criteria,^161^ updated in 2018 with the implementation of mapping techniques.^160^ The Lake Louise criteria consist of a triad combining oedema (as assessed by T2-weighted acquisitions), hyperaemia [reflected by early gadolinium enhancement (EGE)], and necrosis (set by LGE). The presence of ≥2 out of 3 criteria in a suggestive context establishes the diagnosis of myocarditis with high sensitivity and specificity.^144^ Mapping techniques improve intra- and inter-observer diagnostic confidence, the specificity for detecting active inflamamtion and edema, and improve the detection of milder forms of myocarditis.^160^ Additionally, reduced GLS and global circumferential strain could help risk-stratify patients with ICI myocarditis, the magnitude of strain reduction being predictive of MACE.^162^ Nonetheless, the updated Lake Louise criteria might not be as performant in ICI myocarditis. Indeed, recent data show the sensitivity of CMR to be lower in the latter, possibly because of reduced LGE in the early phase.^163,164^ Detecting LGE is particularly challenging in borderline forms of myocarditis,^165^ which display less necrotic insult and patchy distribution. Such patients might benefit from ^18^F-FDG PET, given the increased ^18^F-FDG uptake in myocarditis areas devoid of LGE, which could also guide potential myocardial biopsies.^148^ However, no dedicated study has assessed the diagnostic performance of ^18^F-FDG PET in this specific subgroup. ^18^F-FDG PET could also help distinguish chronic myocarditis from the scarred non-inflammatory myocardium, i.e. healed myocarditis.^166^ Indeed, LGE and strain do not clearly differentiate between chronic and healed myocarditis,^167,168^ whereas ^18^F-FDG uptake decreases in the latter,^166^ a feature that could help monitor treatment response to immunosuppressive therapy.^169^ Given their complementary diagnostic values, studies have evaluated the value of hybrid ^18^F-FDG PET/CMR in myocardial inflammatory diseases,^170,171^ showing an incremental detection of cases with hybrid PET/CMR over single modalities alone.^172^

## Early signs of cardiac dysfunction

**Figure 6:**
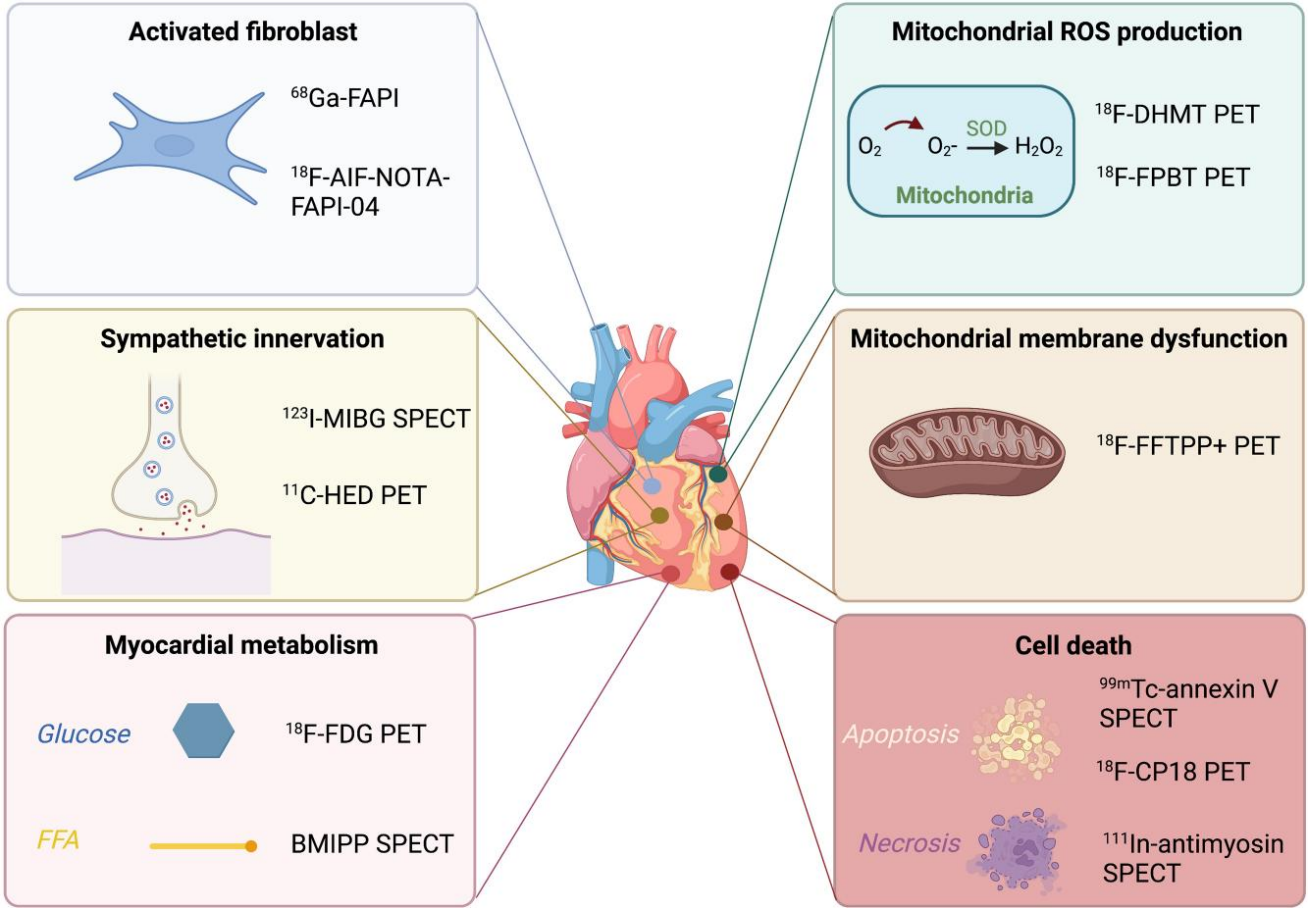
Main metabolic targets of early cardiac toxicity and corresponding radiotracers. Abbreviations: ^11^C-HED, carbon-11-radiolabelled hydroxyephedrine; ^18^F-AIF-NOTA-FAPI-04, fluor-18-labelled 1,4,7-triazacyclononane-N,N′,N″-triacetic acid-conjugated FAP inhibitor 04; ^18^F-CP18, fluor-18-radiolabelled caspase 3 substrate; ^18^F-DHMT, fluor-18-radiolabelled 6-(4-((1-(2-fluoroethyl)-1H-1,2,3-triazol-4-yl)methoxy)phenyl)-5-methyl-5,6-dihydrophenanthridine-3,8-diamine; ^18^F-FDG, fluor-18-radiolabelled fluorodeoxyglucose; ^18^F-FTPP+, fluor-18-radiolabelled (4-fluorophenyl)triphenylphosphonium; ^18^F-FPBT, fluor-18-radiolabelled 3-(3-fluoropropyl)-2-phenyl-2,3-dihydrobenzo[d]thiazole; ^68^Ga-FAPI, gallium-68-radiolabelled fibroblast activation protein inhibitor; ^99m^Tc, technetium-99m; ^111^In, indium-111; ^123^I-MIBG, iodine-123 metaiodobenzylguanidine; BMIPP, beta-methyl-iodine-123 phenylpentadecanoic acid; H_2_O_2_, hydrogen peroxide; O_2_, oxygen; O_2_-, ion oxide; PET, positron emission tomography; ROS, reactive oxygen species; SOD, superoxide dismutase; SPECT, single-photon emission computed tomography.

Numerous efforts aim at detecting early-stage cardiac impairment, i.e. when anticancer treatment is still modifiable or cardioprotective measures can be introduced^8^ (*Figure 6*).

### Cardiac diastolic function

Diastolic dysfunction is a potential early marker of LV dysfunction,^173–175^ which MUGA can assess. MUGA-derived diastolic function parameters include the peak filling rate, time-to-peak filling rate, and first third filling fraction^57,176–178^ that deteriorate before treatment-induced systolic dysfunction.^177,179^ However, the inter- and intra-observer reproducibility of ERNA-based diastolic function is moderate,^57^ questioning its utility in the early detection of CTRCD. CZT-based MUGA is promising, providing a highly reproducible assessment of diastolic function in cancer patients.^180^ Still, MUGA is not routinely used to assess diastolic function, which can easily be obtained from echocardiography.^78^ However, echocardiography is strongly operator-dependent, hampering its interest in surveillance.^74^ CMR can also assess diastolic function based on LV mass and hypertrophy, LA size and function, mitral inflow and pulmonary venous velocity profiles, as well as myocardial deformation imaging with strain. Additionally, T1 mapping and ECV can be used.^181^ CMR presents the advantage over echocardiography of highly reproducible and accurate volume measurements without geometrical or flat profile assumptions.^181^ CMR’s downsides are its restricted availability and the length of sequence acquisitions and image post-processing, limiting its routine use.^181^

### Cardiac sympathetic innervation

Iodine-123 metaiodobenzylguanidine (^123^I-MIBG) reflects the uptake, storage, and release of norepinephrine in the synaptic cleft,^182^ hence allowing cardiac sympathetic innervation imaging.^183^ The main parameter is the heart-to-mediastinum ratio (HMR),^184,185^ i.e. the ratio between cardiac ^123^I-MIBG uptake and a mediastinal reference region of interest. A diminished HMR indicates cardiac sympathetic denervation, either functional (downregulation of post-synaptic β-adrenergic receptors) or due to direct damage (for example, after toxic treatments^186^).

In patients receiving anthracycline, the HMR drops before LVEF,^187,188^ highlighting ^123^I-MIBG’s potential role in early damage detection. Additionally, serial follow-up with cardiac ^123^I-MIBG scintigraphy shows a slight dose-dependent sympathetic impairment following anthracycline administration,^187,189,190^ suggesting a role in damage quantification.

PET radiotracers can also assess cardiac sympathetic activity,^191,192^ notably 6-fluoro-^18^F-L-dihydroxyphenylalanine (^18^F-DOPA), an analogue of L-dihydroxyphenylalanine (L-DOPA) routinely used to investigate neuroendocrine tumours.^193^ Another norepinephrine analogue is carbon-11-radiolabelled hydroxyephedrine (^11^C-HED),^194^ in which the need for on-site production limits clinical use. To date, however, no study has specifically studied these radiotracers to diagnose CTRCD.

### Myocardial metabolism

Cardiac metabolism is a balance between various fuels, depending on the substrate’s bloodstream availability, dietary conditions, and underlying myocardial conditions.^195^ Under physiologic conditions, free fatty acids (FFAs) and glucose represent the primary cardiac energy sources.^196^ Myocardial glucose consumption can be imaged with ^18^F-FDG and FFA uptake with beta-methyl-iodine-123 phenylpentadecanoic acid (BMIPP).^197^ In the fasting phase, FFAs are abundantly available to the heart,^196^ rendering BMIPP more advantageous for assessing cardiac metabolism than ^18^F-FDG.^198^ Nonetheless, BMIPP is only routinely used in Japan,^199^ and one study reported BMIPP uptake reduction in patients receiving taxanes.^200^ Conversely, ^18^F-FDG PET is largely available and part of the routine oncological assessment. In patients treated with doxorubicin, an increased LV ^18^F-FDG uptake from baseline to end-of-treatment PET is associated with a subsequent LVEF drop^85^ and MACE.^201^ Moreover, increased RV ^18^F-FDG uptake predicts a higher cardiotoxicity risk.^202^ Similarly, in chest radiotherapy patients, focal cardiac ^18^F-FDG uptake is associated with myocardial damage,^203–205^ a study pointing towards a relation between the radiotherapy dose and the intensity of ^18^F-FDG uptake.^206^ Focal ^18^F-FDG cardiac uptake in cancer patients correlates highly with perfusion abnormalities on SPECT MPI,^207^ giving potential mechanistic insights for the subsequent cardiotoxicity. Still, a significant drawback of ^18^F-FDG PET is the high variability of cardiac uptake with diet and insulinaemia,^208^ which could be reduced by prolonged fasting.^209^ Additionally, ^18^F-FDG myocardial uptake increases in the ischaemic myocardium, which, although limiting the specificity of ^18^F-FDG patterns, could identify ischaemia onset.^210^

Alternatively, carbon-11 (^11^C) radiotracers can be used to image myocardial metabolism. ^11^C-acetate is taken up by cardiomyocytes and converted to acetyl-CoA, a substrate for energy production via the tricarboxylic acid cycle.^211^ The rate of ^11^C-acetate uptake is a marker of myocardial oxidative metabolism.^212^ In a pre-clinical model of mice undergoing treatment by tyrosine kinase inhibitors, the myocardium showed a decrease in ^11^C-acetate uptake concomitantly to an increase in ^18^F-FDG uptake.^213^ The short half-life of ^11^C (∼20 min), although interesting from a radiation exposure perspective, is the main factor limiting its routine use, as ^11^C requires an on-site cyclotron.^211^

### Mitochondrial metabolism

The bottleneck of all cellular energy pathways is the mitochondrial production of ATP. Several chemotherapies affect ATP production and lead to cell death, generally by increasing reactive oxygen species (ROS) production.^198^ A PET radiotracer targeting ROS has recently been developed, named ^18^F-6-(4-((1-(2-fluoroethyl)-1H-1,2,3-triazol-4-yl)methoxy)phenyl)-5-methyl-5,6-dihydrophenanthridine-3,8-diamine (^18^F-DHMT). In a pre-clinical rodent model of anthracycline-induced cardiotoxicity, ^18^F-DHMT evidenced an increased myocardial ROS production before any LV drop.^214^ Another ROS-targeting radiotracer is ^18^F-3-(3-fluoropropyl)-2-phenyl-2,3-dihydrobenzo[d]thiazole (^18^F-FPBT), in which myocardial uptake is also increased in rats receiving anthracycline.^215^ Deregulation of cardiomyocyte homeostasis by chemotherapy can manifest as mitochondrial membrane dysfunction, which can be explored with ^18^F(4-fluorophenyl)triphenylphosphonium (^18^F-FTPP+). In a swine model receiving intracoronary infusions of anthracycline, ^18^F-FTPP+ showed a partial mitochondrial depolarization in myocardial areas distal to the infused vessel.^216^ Recently, a radiotracer targeting TSPO, a translocator protein expressed in mitochondrial-activated microglia, has been validated in a model of myocardial infarction.^217^ This pre-clinical study showed that an early myocardial uptake of ^18^F-radiolabelled TSPO on PET predicted the subsequent LVEF reduction.

### Cell death

A hallmark apoptosis feature is the exposition of phosphatidylserine at the cellular surface.^218^ Technetium-99m (^99m^Tc)-radiolabelled annexin V is a phosphatidylserine ligand that detects apoptotic cardiomyocytes.^219^ In rats receiving doxorubicin, ^99m^Tc-radiolabelled annexin V evidenced drug-induced toxicity in a dose-dependent manner before any functional impairment on echography.^220^ Recently, PET apoptosis radiotracers have also been developed.^221^ In a mouse model of experimentally induced anthracycline cardiotoxicity, ^18^F-CP18, a substrate of the caspase 3 enzyme present in apoptotic cells,^222^ evidenced apoptosis before any LVEF drop.^223^ Another target is myosin, externalized by necrotic cells after membrane rupture. Preliminary clinical studies showed that increased myocardial uptake of an indium-111 (^111^In)-radiolabelled antimyosin antibody preceded LVEF modifications in patients receiving anthracycline.^189,224,225^

### Myocardial fibrosis

The cardiomyocyte loss induced by anticancer treatments is accompanied by myocardial fibroblast activation, leading to fibrotic ventricular remodelling, a condition of increased risk for heart failure.^226^ Although echocardiography and CMR can detect cardiac fibrosis, even at an early stage with mapping techniques,^227,228^ fibrosis still indicates myocardial damage. Therefore, detecting the onset of fibrotic replacement could help initiate cardiac treatments at an early and reversible stage.^227^ Fibroblast activation protein (FAP) is a transmembrane protease with enhanced expression in activated fibroblasts.^229^ Recently, pre-clinical findings evidenced intense ^68^Ga-FAPI myocardial uptake in areas of activated fibroblasts, conversely to no uptake in areas of advanced fibrosis.^230–232^ Similar incidental cases of ^68^Ga-FAPI cardiac uptake have been reported in cancer patients, unveiling myocardial ischaemia.^233^ This suggests that ^68^Ga-FAPI PET, likely to be used for cancer staging, could help simultaneously detect early stages of myocardial fibrosis. Moreover, ^68^Ga-FAPI myocardial uptake could pre-date any LVEF decrease, suggesting a potential role in cardiotoxicity prediction.^234^ Similarly, an ^18^F-radiolabelled FAPI tracer (^18^F-AlF-NOTA-FAPI-04) detects radiation-induced myocardial ischaemia before LVEF decreases, comforting the potential role of FAPI radiotracers for the early identification of cardiac damage.^235^

## Future directions

One next step is to stratify the cardiotoxicity risk before treatment initiation. Predictive scores based on CVRFs and biological markers^94,236,237^ could be augmented by non-invasive imaging. For example, myocardial ^18^F-FDG uptake obtained from routine staging ^18^F-FDG PET can help stratify the cardiovascular risk with no additional cost or radiation burden.^207^ Cardiovascular risk stratification could also benefit from hybrid PET/CMR by combining CMR mapping techniques with the prognostic value of myocardial ^18^F-FDG uptake to predict the MACE risk.^238,239^

Artificial intelligence (AI) is a potential game changer in cardio-oncology.^240,241^ In 2619 cancer-free patients explored with SPECT MPI, a machine learning analysis combined with clinical data outperformed human analysis for MACE prediction.^242^ Moreover, the higher reproducibility of machine learning could improve diagnostic confidence in uncertain myocarditis patterns, such as patchy ^18^F-FDG myocardial uptake. AI also improves the characterization of several types of malignant masses,^243–245^ which might benefit cardiac tumour characterization.

In the era of precision medicine, where similar phenotypes arise from different genomic, metabolomic, and proteomic profiles, it will be crucial to tailoring the diagnosis to the tumour’s ‘-omic signature’.^246^ As a metabolic tool targeting specific pathophysiological pathways, nuclear imaging will most certainly play a central role in precision cardio-oncology.^246^ In addition to mapping cardiotoxicity, these probes might play a theranostic role, as with SSTR radiotracers, which help select patients in whom peptide receptor radionuclide therapy is indicated.^247^ An unsuccessful attempt in this sense has been made with ^111^In-labelled trastuzumab scintigraphy to predict cardiotoxicity from trastuzumab.^248^ Yet, the theranostic field is still in its infancy, and the wideness of metabolic targets assessable with nuclear radiotracers renders this goal within reach.

## Conclusion

The progress in anticancer treatment is progressively turning cancer into a chronic condition. Consequently, the new challenge in this population is slowly shifting towards tackling other mortality causes, particularly CVD. Nuclear imaging allows for diagnosing various cardiac complications of anticancer therapies, even at an early stage, is useful for disease monitoring, and is a promising tool for the risk stratification of patients receiving cardiotoxic treatments. In addition, nuclear imaging has the unique ability to target specific metabolic links in the cardiotoxicity cascade for either diagnosis or treatment. Leveraging radiotracers already used routinely in patients with cancer, such as ^18^F-FDG and MPI tracers, could benefit this population with no additional cost or radiation exposure. Consequently, in the expanding field of cardio-oncology, nuclear medicine remains a central player that will most certainly remain at the forefront of the diagnostic armamentarium alongside cross-sectional imaging.

## Data Availability

No new data were generated or analysed in support of this research.
